# Supplementary material for: An in vivo Comparison Study Between Strontium Nanoparticles and rhBMP2
Source: Front Bioeng Biotechnol. 2020 Jun 16;8:499. doi: 10.3389/fbioe.2020.00499 (PMC7308719; doi:10.3389/fbioe.2020.00499)
Supplement: Supplementary file 1 [file Presentation_1.pptx]

## Slide 1
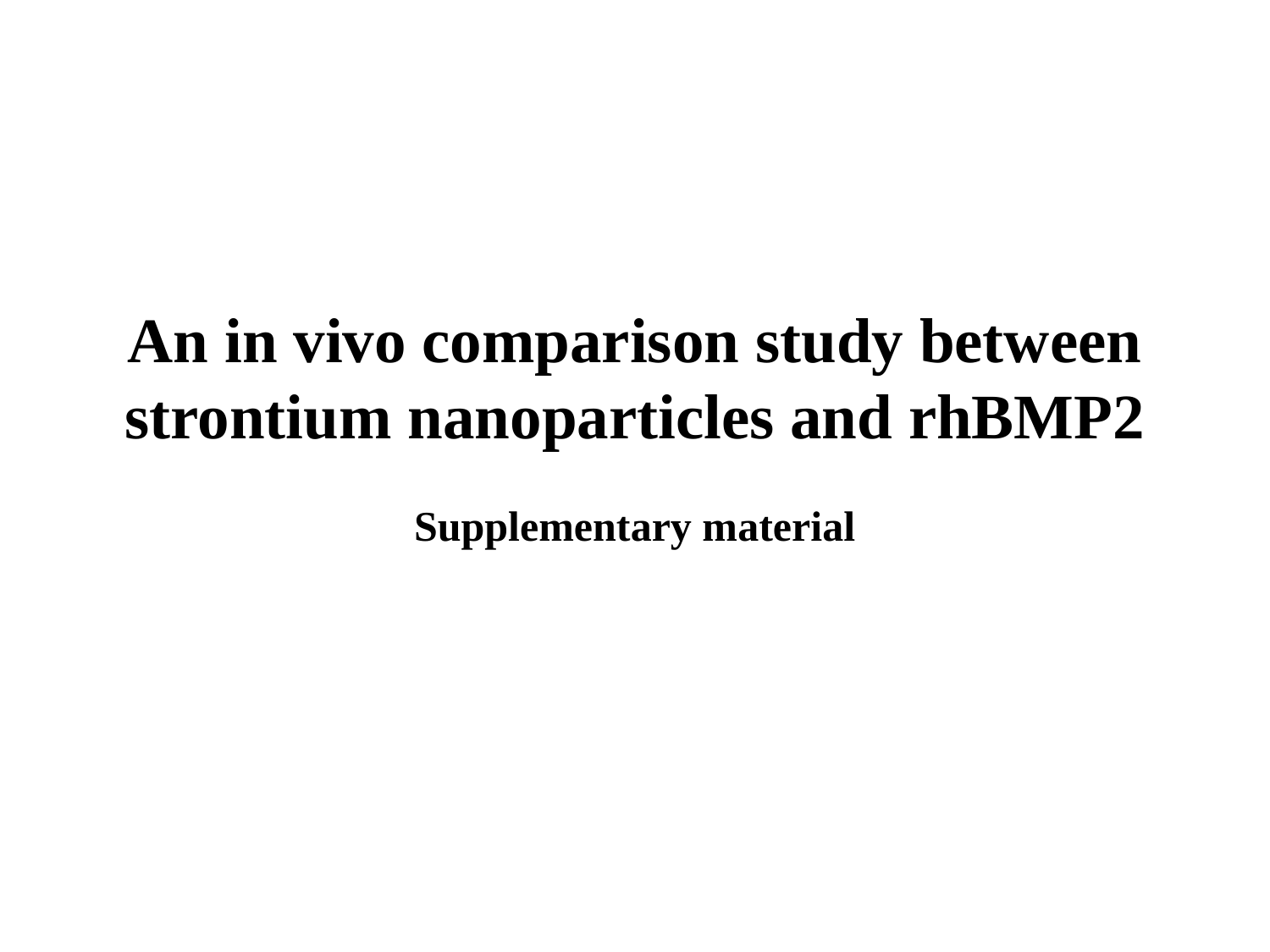

An in vivo comparison study between strontium nanoparticles and rhBMP2
Supplementary material

## Slide 2
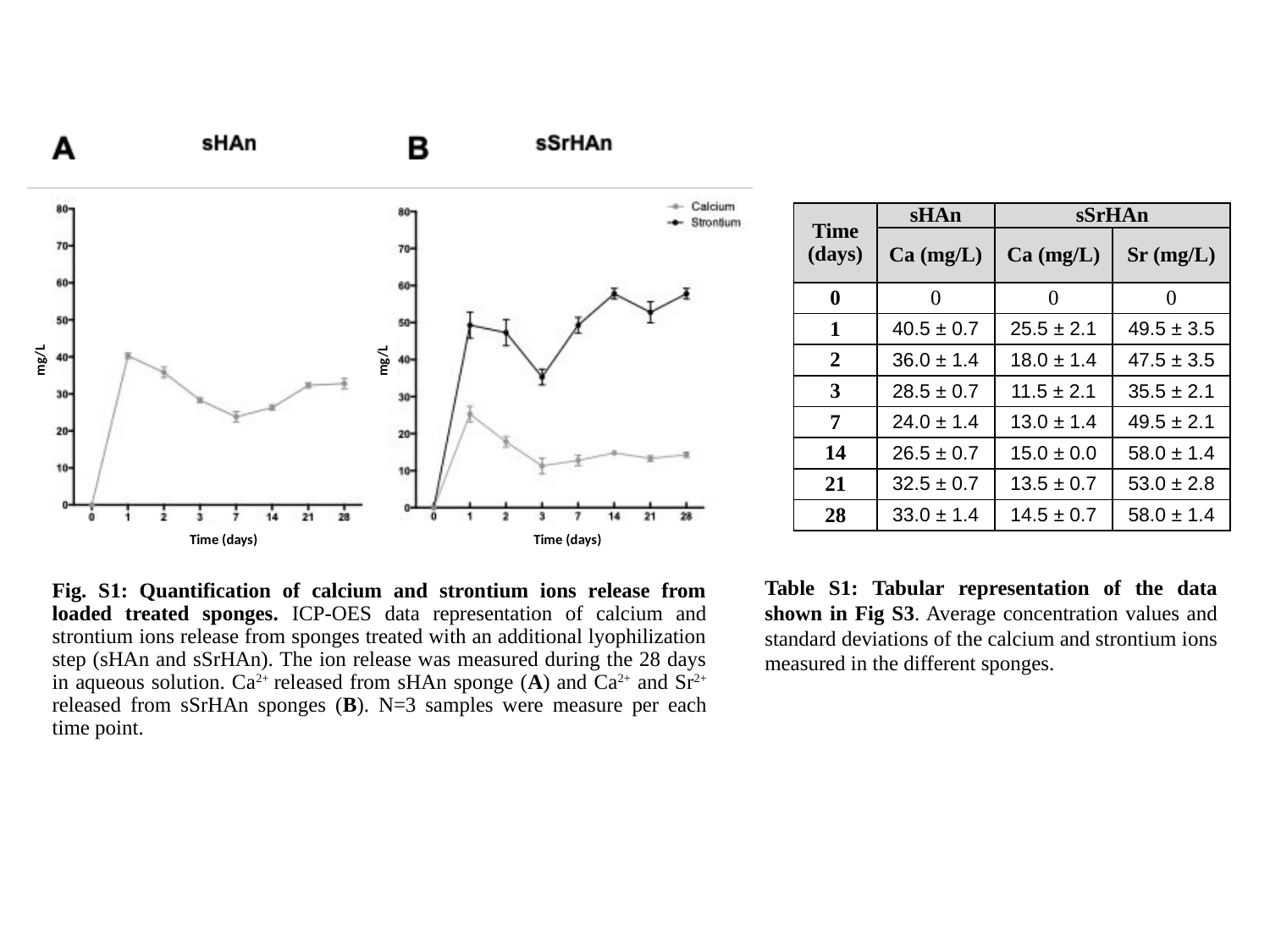

mg/L
mg/L
Time (days)
Time (days)
| Time (days) | sHAn | sSrHAn | |
| --- | --- | --- | --- |
| | Ca (mg/L) | Ca (mg/L) | Sr (mg/L) |
| 0 | 0 | 0 | 0 |
| 1 | 40.5 ± 0.7 | 25.5 ± 2.1 | 49.5 ± 3.5 |
| 2 | 36.0 ± 1.4 | 18.0 ± 1.4 | 47.5 ± 3.5 |
| 3 | 28.5 ± 0.7 | 11.5 ± 2.1 | 35.5 ± 2.1 |
| 7 | 24.0 ± 1.4 | 13.0 ± 1.4 | 49.5 ± 2.1 |
| 14 | 26.5 ± 0.7 | 15.0 ± 0.0 | 58.0 ± 1.4 |
| 21 | 32.5 ± 0.7 | 13.5 ± 0.7 | 53.0 ± 2.8 |
| 28 | 33.0 ± 1.4 | 14.5 ± 0.7 | 58.0 ± 1.4 |
Table S1: Tabular representation of the data shown in Fig S3. Average concentration values and standard deviations of the calcium and strontium ions measured in the different sponges.
Fig. S1: Quantification of calcium and strontium ions release from loaded treated sponges. ICP-OES data representation of calcium and strontium ions release from sponges treated with an additional lyophilization step (sHAn and sSrHAn). The ion release was measured during the 28 days in aqueous solution. Ca2+ released from sHAn sponge (A) and Ca2+ and Sr2+ released from sSrHAn sponges (B). N=3 samples were measure per each time point.

## Slide 3
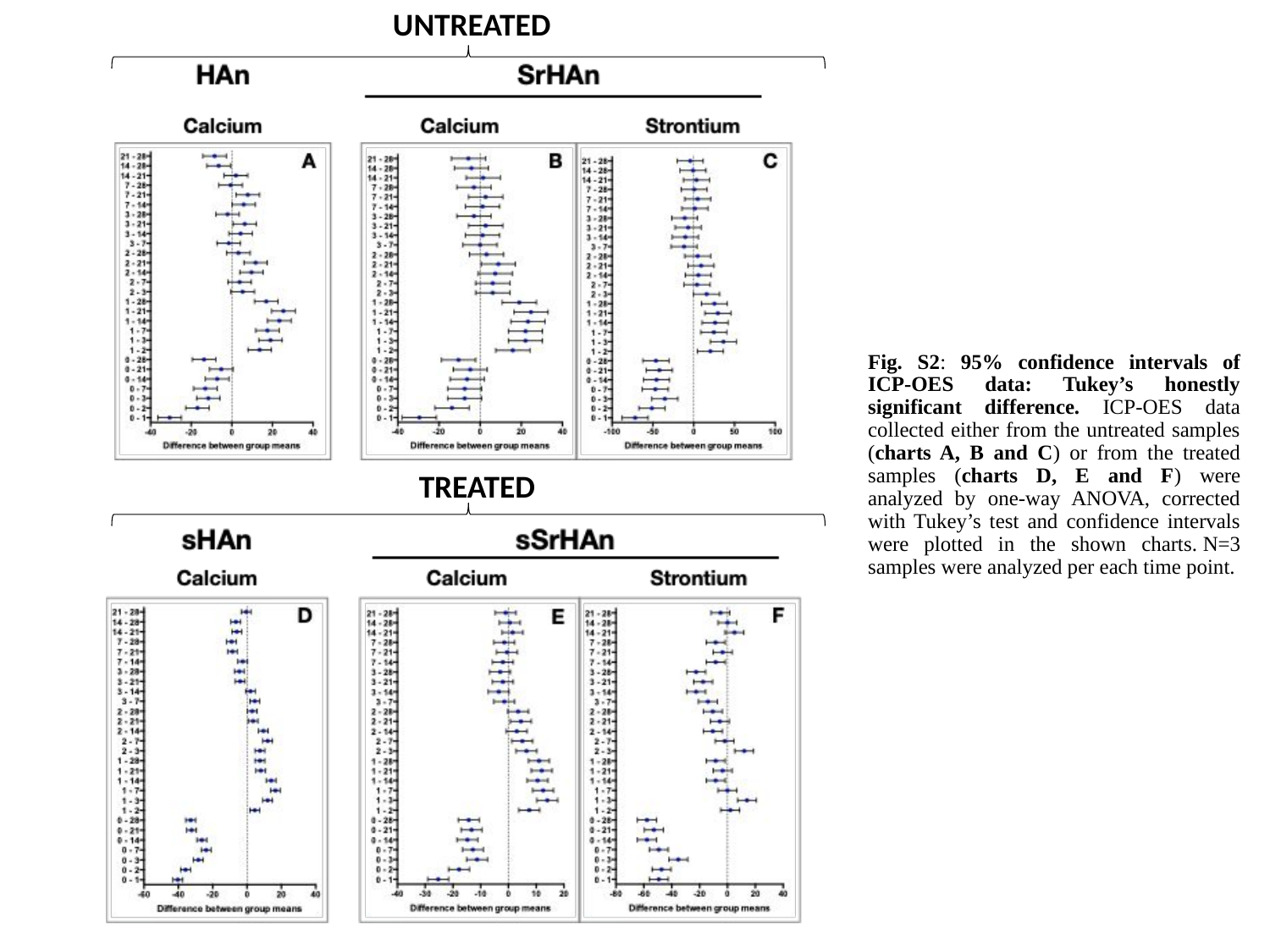

UNTREATED
Fig. S2: 95% confidence intervals of ICP-OES data: Tukey’s honestly significant difference. ICP-OES data collected either from the untreated samples (charts A, B and C) or from the treated samples (charts D, E and F) were analyzed by one-way ANOVA, corrected with Tukey’s test and confidence intervals were plotted in the shown charts. N=3 samples were analyzed per each time point.
TREATED

## Slide 4
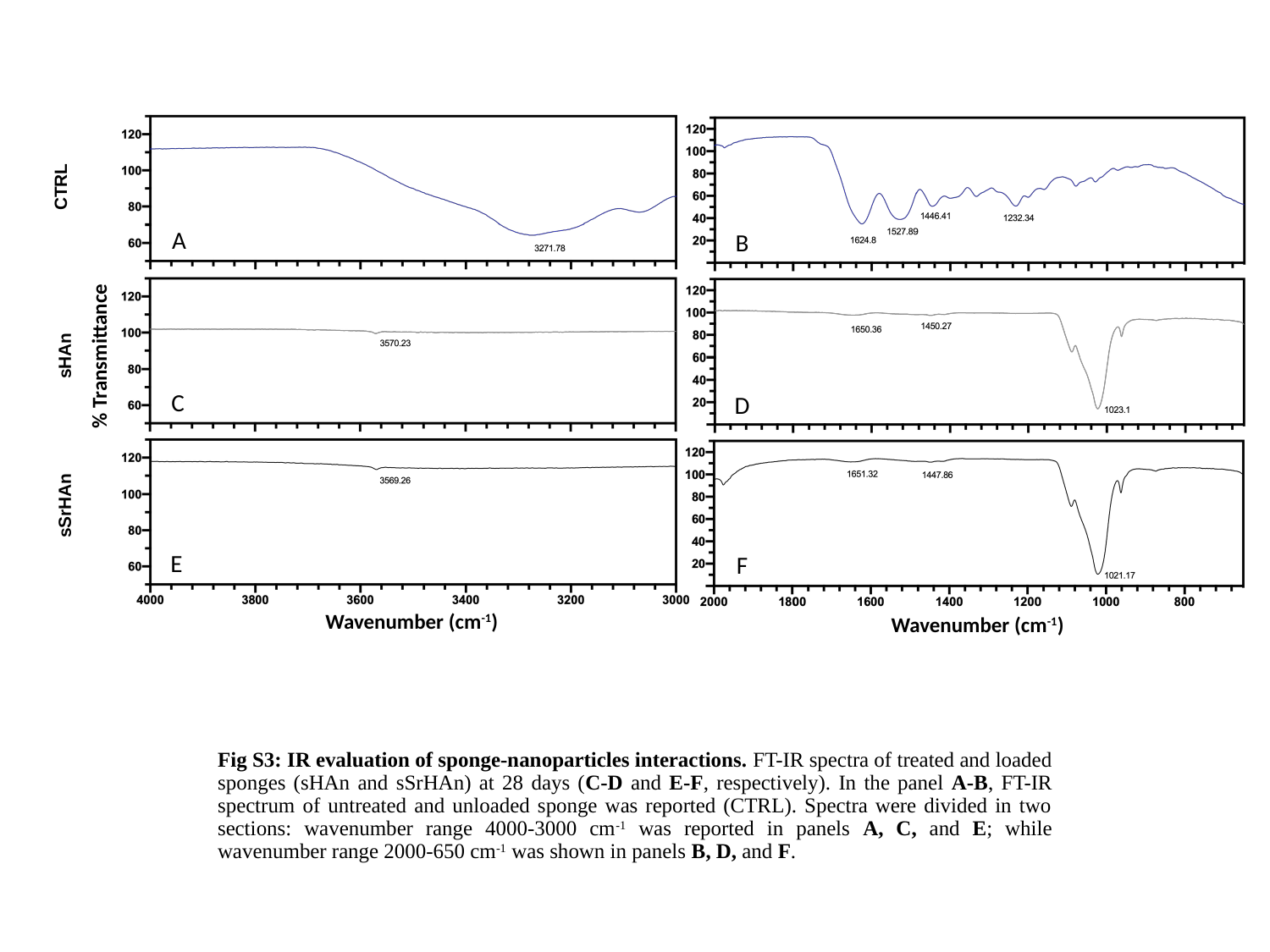

CTRL
A
B
% Transmittance
sHAn
C
D
sSrHAn
E
F
Wavenumber (cm-1)
Wavenumber (cm-1)
Fig S3: IR evaluation of sponge-nanoparticles interactions. FT-IR spectra of treated and loaded sponges (sHAn and sSrHAn) at 28 days (C-D and E-F, respectively). In the panel A-B, FT-IR spectrum of untreated and unloaded sponge was reported (CTRL). Spectra were divided in two sections: wavenumber range 4000-3000 cm-1 was reported in panels A, C, and E; while wavenumber range 2000-650 cm-1 was shown in panels B, D, and F.

## Slide 5
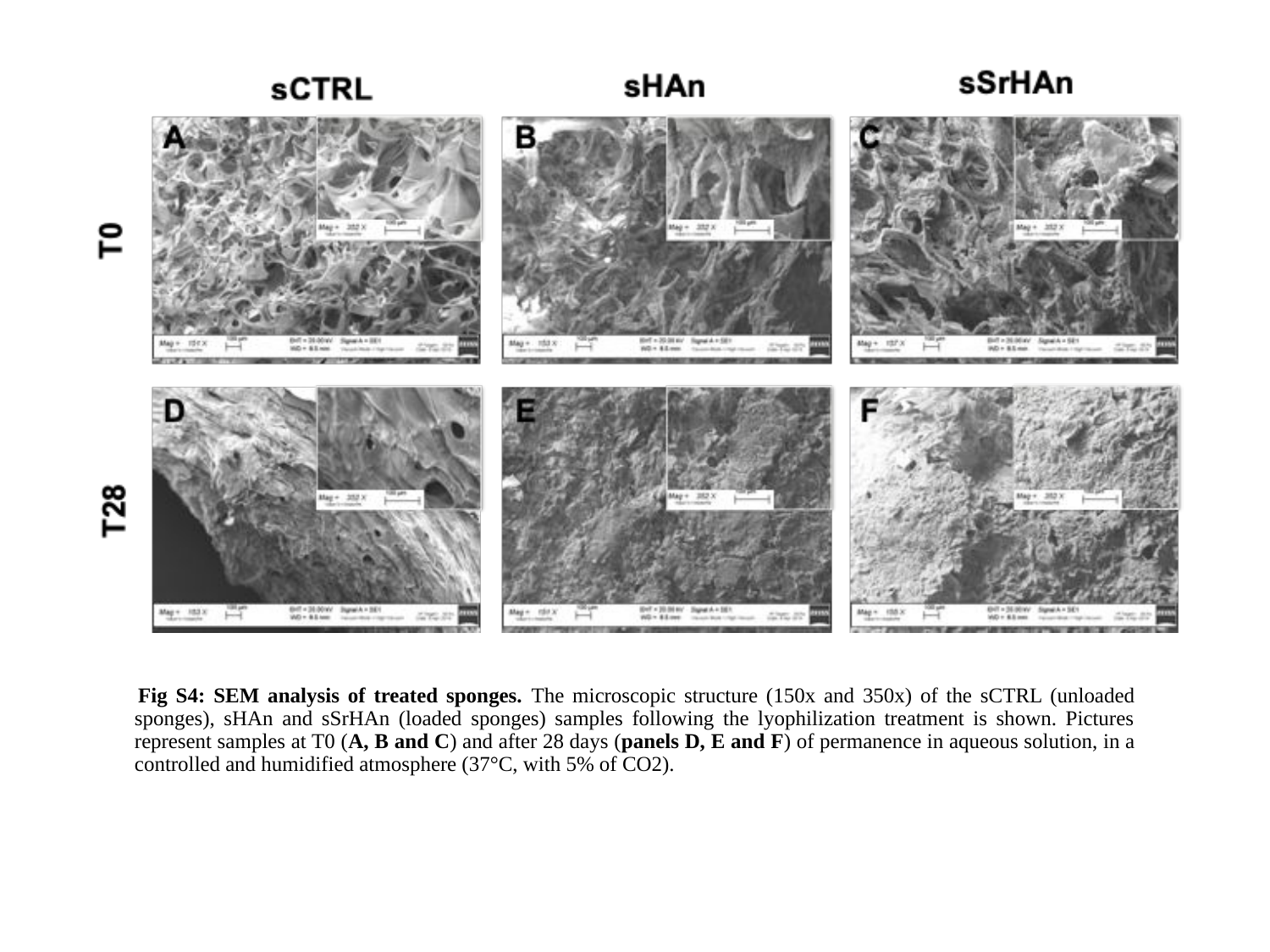

Fig S4: SEM analysis of treated sponges. The microscopic structure (150x and 350x) of the sCTRL (unloaded sponges), sHAn and sSrHAn (loaded sponges) samples following the lyophilization treatment is shown. Pictures represent samples at T0 (A, B and C) and after 28 days (panels D, E and F) of permanence in aqueous solution, in a controlled and humidified atmosphere (37°C, with 5% of CO2).
